# Design and Development of a Lead-Freepiezoelectric Energy Harvester for Wideband, Low Frequency, and Low Amplitude Vibrations

**DOI:** 10.3390/mi12121537

**Published:** 2021-12-10

**Authors:** Neetu Kumari, Micky Rakotondrabe

**Affiliations:** 1 Department of Automatic Control and Micro-Mechatronic Systems, FEMTO-ST Institute, Université Bourgogne Franche-Comté, CNRS, 24 rue Alain Savary, 25000 Besançon, France; 2 LGP Laboratory, National School of Engineering in Tarbes (ENIT-INPT), University of Toulouse, 65000 Tarbes, France

**Keywords:** vibrational piezoelectric energy harvesting, multiple and low frequency, lead-free lithium niobate material, nonlinearity, shape memory alloy

## Abstract

In recent years, energy harvesting from ambient vibrations using piezoelectric materials has become the center of attention due to the fact that it has the potential to replace batteries, providing an easy way to power wireless and low power sensors and electronic devices. Piezoelectric material has been extensively used in energy harvesting technologies. However, the most commercially available and widely used piezoelectric materials are lead-based, Pb$\;\left[Zr_{x}Ti_{1-x}\right]$ $O_{3}\;$(PZT), which contains more than 60 weight percent lead (Pb). Due to its extremely hazardous effects on lead elements, there is a strong need to substitute PZT with new lead-free materials that have comparable properties to those of PZT. Lead-free lithium niobate (LiNbO_3_) piezoelectric material can be considered as a substitute for lead-based piezoelectric materials for vibrational energy scavenging applications. LiNbO_3_ crystal has a lower dielectric constant comparison to the conventional piezoceramics (for instance, PZT); however, at the same time, LiNbO_3_ (LN) single crystal presents a figure of merits similar to that of PZT, which makes it the most suitable choice for a vibrational energy harvester based on lead-free materials. The implementation was carried out using a global optimization approach including a thick single-crystal film on a metal substrate with optimized clamped capacitance for better impedance matching conditions. A lot of research shows that standard designs such as linear piezoelectric energy harvesters are not a prominent solution as they can only operate in a narrow bandwidth because of their single high resonant peak in their frequency spectrum. In this paper, we propose, and experimentally validate, a novel lead-free piezoelectric energy harvester to harness electrical energy from wideband, low-frequency, and low-amplitude ambient vibration. To reach this target, the harvester is designed to combine multi-frequency and nonlinear techniques. The proposed energy harvesting system consists of six piezoelectric cantilevers of different sizes and different resonant frequencies. Each is based on lead-free lithium niobate piezoelectric material coupled with a shape memory alloy (nitinol) substrate. The design is in the form of a circular ring to which the cantilevers are embedded to create nonlinear behavior when excited with ambient vibrations. The finite element simulation and the experimental results confirm that the proposed lead-free harvester design is efficient at low frequencies, particularly different frequencies below 250 Hz.

## 1. Introduction

Over the years, harvesting vibration, which is present in the form of kinetic energy in our surroundings to generate electricity, has gained a lot of attention due to its promising potential in powering miniaturized and low power consumption devices [1,2,3]. A lot of research has been carried out based on different harvester’s mechanisms or principles of conversion, such as electromagnetic [4,5], electrostatic [6], piezoelectric [7,8,9,10,11], or hybrid piezoelectric energy harvesters [12]. In the literature, the piezoelectric effect is among, if not the first, most employed principle in the field of mechanical micro energy conversion [13,14,15]. Based on the “direct piezoelectric effect” phenomenon that is also employed in sensors [16] and self-sensing actuators [17,18] for further feedback control [19,20,21,22], piezoelectric energy harvesting devices can scavenge the energy from vibrations and motion present in the surroundings to provide the maximum output voltage when operating at their resonance frequencies. However, exciting the devices at their resonance frequencies is challenging because the available surrounding frequency is generally low whilst the resonance of the harvester’s structure is high. It is therefore of paramount importance to widen the spectrum and to include lower frequencies when designing vibrational piezoelectric energy harvester (VPEH) devices so that they can be used in real applications and situations. To this aim, investigations have been undertaken on different variants of the vibrational piezoelectric energy harvester (VPEH), including linear and nonlinear oscillators [23,24], multiscale [25,26,27], or millidegrees of freedom designs [28,29], or VPEHs working with stochastic excitation [30,31,32]. A nonlinear technique used to widen and lower the resonance frequencies employs magnets to enable the bistable functioning of the VPEH [33]. The advantage of bistable designs over linear VPEHs is that they are less dependent on the frequency of excitation but require only an external force to pass from one stable state to another. In contrast, when the excitation force is low, one cannot activate the bistable functioning, and thus the overall functioning, of the VPEH. This activation threshold strongly depends on the materials used, the geometry, and the boundary. Recent work [34] proposed a more efficient snap-through solution reducing the activation force, but the required threshold is still high for certain applications. A mechanical approach to VPEH design for low-frequency application is the addition of a mass at the tip of the structure, which reduces its resonance [35]. However, this approach is only used for one single frequency since one has to modify the mass, or its placement, to fit the structure for a different excitation frequency, which is consequently infeasible for miniaturized VPEHs. It is therefore important to find the optimal shape of the VPEH structures such that they fit with prescribed low frequency. For instance, systematic design techniques based on topology optimization [36,37], or based on interval techniques [38], have also been used in [39,40], whilst a genetic algorithm was used in [41]. However, these works concern linear structures and their excitation forces, and thus the amplitude of vibration was supposed to be sufficiently high. To harvest energy from the surroundings with a wide and low frequency range and low amplitude vibration, we propose a new multimodal piezoelectric energy harvester MPEH structure design based on nonlinear functioning. The proposed design is based on a circular ring embedded in six cantilevers of different resonance frequencies and energy conversions. While the entire structure exhibits nonlinear functioning, allowing functioning at low amplitude vibrations, we propose a lead-free material (single-crystal lithium niobate LiNbO3) as the piezoelectric layer and a shape memory alloy as the substrate. Finite element simulation and experiments on the fabricated design are carried out and validate the functioning of the MPEH with the expected conditions, i.e., low amplitude, wide and low frequency range. The fact that the material used is lead-free makes this harmless and thus ensures that the MPEH is utilizable for daily life applications such as powering elder people’s tracking sensors, watches and electronic wearables, and autonomous sensors in vehicles. The remainder of the paper is organized as follows. Here we are targeting to place this transducer inside automobiles to scavenge energy from the unwanted vibration occurrences in the environment. We present in Section 2 the new MPEH design. Section 3 is devoted to a simulation study. In Section 4, we present the experimental setup and the experimental results regarding the MPEH. Finally, conclusions and perspectives are given in Section 5.

## 2. Design of a Multi-Frequency Piezoelectric Energy Harvester

**Figure 1:**
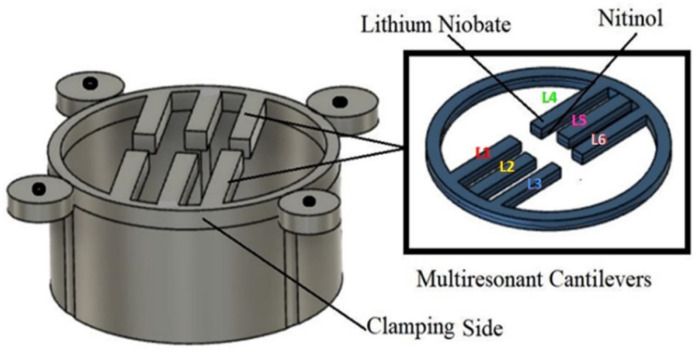
Schematic of the proposed multi resonant piezoelectric energy harvester.

The proposed nonlinear piezoelectric energy harvesting system of this study is shown in Figure 1. This energy harvesting system is made of six cantilever beams and a monobloc with a circular ring. The monobloc ring and six cantilevers have two layers: a lithium niobate (LiNbO_3_, 127.8° Y-cut, from Roditi systems Inc.;London, UK) layer, which is a lead-free piezoelectric material, and a shape memory alloy (SMA) based on the nitinol material (composition is 55:45 Ni: Ti, from Nexametals company, Ogun, Nigeria) which serves as a passive layer. To fabricate the structure, the first step consists of separately cutting down a LiNbO_3_ wafer and an SMA wafer to obtain the desired shape (circular ring with six cantilevers) for each. The cutting process is conducted using a femtosecond laser cutting machine with several distinct advantages: high resolutions (down to 25 nm), noncontact interaction, and can be applied to any substrate without specific conditioning [42,43]. After getting the desired shape, both samples were bonded together using silver glue with epoxy and were kept in an autoclave for curing at 120 °C for one hour thirty minutes. To add the electrodes, a mask is placed, and gold sputtering is conducted, on the Nitinol and lithium niobate on the top and bottom surface. The six cantilever beams are of different lengths but have the same thickness and width. However, all six cantilevers are connected to the same electrodes to generate more electrical power than an individual consideration. An important and attractive feature of this proposed energy harvester compared to the others described in the literature is that there is no need to apply the traditional tip mass to reach a relatively low frequency (less than 500 Hz). Indeed, the chosen materials (SMA combined with LiNbO_3_) with appropriate dimensions and the nonlinearity (embedding on a circular shape) allow the proposed structure to work at a low frequency and low amplitude of vibration whilst the six cantilevers of different sizes allow a wide range of principal working frequency (six resonance frequencies).

Finally, another feature of this proposed structure is its adaptability and simplicity of design: its form is easy to fabricate, duplicate, or modify, for instance, if one needs to put additional cantilevers to increase the frequency range and the output power. The circular ring of the design has an outer diameter of 55 mm and an inner diameter of 50 mm. The choice of the cantilevers’ dimensions allows the tuning of their resonance frequencies. Thus, to make the MPEH system performant for further analysis, we used finite element analysis (COMSOL Multiphysics software 5.5, Burlington, MA, USA) to optimize the cantilever beam’s geometry. We considered the parameters of the design along with the cantilever beam’s material properties. Indeed, the material properties and geometry have an essential role to play as these properties affect the vibration response of the multiresonant piezoelectric energy harvester, including the system’s resonance frequency. A full parametric study was therefore completed to find the cantilever beams’ optimum lengths (L1, L2, L3, L4, L5, and L6) using COMSOL Multiphysics, as presented in the next section. This study’s primary focus is to target a frequency range between 1 Hz and 500 Hz. We want to use this device in automobiles to power sensors present in them, as in those environments, the vibrating frequency is usually less than 500 Hz. The objective of this investigation is to optimize the geometry and determine the lowest resonance frequency without using the proof mass. The resonance frequency can be further tuned if we desire to implement the proof mass. The proposed multiresonant piezoelectric energy harvester’s fabricated prototype was experimentally tested under harmonic base excitation, as presented in the following section.

## 3. Finite Element Analysis of Multiresonant Piezoelectric Energy Harvester

We used COMSOL Multiphysics 5.5 software with a “Piezoelectric Module” for the FEA study [44]. The total geometry, including the ring with all six attached cantilevers, was modelled in 3D. Both of the materials, lithium niobate and the shape memory alloy (Nitinol), were selected from the material library. The simulation evaluated the bending, compression, and shear mode behaviors of the beam. The material properties of the lithium niobate and the Nitinol were the same, as shown in Table 1. The ring was kept at the fixed constraints for the boundary conditions when performing the simulation, and the six cantilevers were selected as free. During the meshing in COMSOL Multiphysics software, the elements’ size was chosen as 0.8 mm, getting accurate results with minimum simulation time. Acceleration applied to the system was controlled at 1 g. From the simulation, we can check the device’s resonance frequencies and the output voltage achieved from the device. We know that the strain is directly related to the output voltage, therefore, the higher the stress, the higher the output voltage will be. According to the literature [45], connecting different energy harvesting cantilever beams of the same thickness but with different lengths, can produce higher output power. It was reported that the output power was increased from 2 μW to 5 μW, and the bandwidth was widened from (47, 55) Hz to (22, 88) Hz. Therefore, in this study, we decided to use the same thickness but different length cantilevers. In the next section, we present the results of the static structural analysis obtained from FEA. We compared the stress, strain, and deflection of the cantilever while changing the cantilever’s length. An eigenfrequency analysis was also carried out to find the modes present for the device.

### 3.1. FEA Mathematical Modeling 

As seen in [46], the constitutive equations of linear piezoelectric material are presented in Equation (1), which represents the material behavior and is used in the FEM software for simulation.

(1) $$T=c_{E}S-eE$$

$$D=e^{T}S+\varepsilon_{S}E$$

where *T* is the stress vector, *D* is the electric flux density vector, *S* is the strain vector, *E* is the electric field vector, *c_E_* is the elasticity matrix (evaluated at the constant electric field), *e^T^* is the piezoelectric stress matrix, and *ε_S_* is the dielectric matrix (estimated at constant mechanical strain). Here, Equation (2) represents the material behavior which the FEM software solves. The finite element discretization is performed by establishing nodal solution variables and element shape functions over an element domain which approximates the following solution:

(2) $$u_{c}=N_{u}^{T}.u$$

$$V_{c}\;=\;N_{V}^{T}.V$$

where *u_c_* is the displacement within the element domain in the x, y, z directions, *V_c_* is the electrical potential within the element domain, *N_u_* is the matrix of displacement shape functions, *N_V_* is the vector of the electrical potential shape function, *u* is the vector of nodal displacements, and *V* is the vector of nodal electrical potential. Using Equation (3), the strain *S* and electric field E are thus related to the displacement and potential of Equations (4) and (5), respectively. Consider

(3) $$S=B_{u}.u$$

(4) $$E=-B_{V}.V$$

where:

(5) $$Bu=\left[\begin{array}{cccccc} \frac{\partial }{\partial x} & 0 & 0 & \frac{\partial }{\partial y} & 0 & \frac{\partial }{\partial z}\\ 0 & \frac{\partial }{\partial y} & 0 & \frac{\partial }{\partial x} & \frac{\partial }{\partial z} & 0\\ 0 & 0 & \frac{\partial }{\partial z} & 0 & \frac{\partial }{\partial y} & \frac{\partial }{\partial x} \end{array}\right]$$

$$B_{y}={\left[\begin{array}{ccc} \frac{\partial }{\partial x} & \frac{\partial }{\partial y} & \frac{\partial }{\partial z} \end{array}\right]}^{T}$$

After implementing the finite element discretization, the coupled finite element matrix equation is given:

(6) $$\left[\begin{array}{cc} M & 0\\ 0 & 0 \end{array}\right]\left[\begin{array}{c} \ddot{u}\\ \ddot{V} \end{array}\right]+\left[\begin{array}{cc} C & 0\\ 0 & 0 \end{array}\right]\left[\begin{array}{c} \ddot{u}\\ \ddot{V} \end{array}\right]+\left[\begin{array}{cc} K & K_{z}\\ K_{z}^{T} & K_{d} \end{array}\right]\left[\begin{array}{c} u\\ V \end{array}\right]=\left[\begin{array}{c} F\\ L \end{array}\right]$$

(7) $$M=\int \rho N_{u}N_{u}^{T}dv$$

The damping matrix (*C*) may be used in harmonic, damped modal, and transient analyses and substructure generation. In its most general form, it is given by Equation (8):

(8) $$M=\alpha M+\left(\beta +\beta_{c}\right)K+\overset{N_{m}}{\underset{j=1}{\sum }}\left[\left(\beta_{j}^{m}+\frac{2}{Ω}\beta_{j}^{\xi }\right)K_{j}\right]+\overset{N_{e}}{\underset{k=1}{\sum }}C_{k}+C_{\xi }$$

where: $\beta_{j}^{\xi }$ is frequency-independent (constant stiffness matrix coefficient for material *j*, Ω—circular excitation frequency); *K_j_* is the portion of structure stiffness matrix based on material *j*; *Ne* is the number of elements with specified damping (*C_k_*—element damping matrix, *C_ξ_*—frequency-dependent, damping), *C* is the structural damping matrix; *a* is the mass matrix multiplier; *M* is the structure mass matrix; *β* is the stiffness matrix multiplier; *β_c_* is the variable stiffness matrix multiplier; *K* is the mechanical structure stiffness matrix; and *N_m_* is the number of materials with $\beta_{j}^{m}\;$(stiffness matrix multiplier for material *j*).

For structural analysis, one of the main parameters is mechanical structural stiffness. This is a matrix method that makes use of the members’ stiffness relations for computing member forces and displacements in structures and is depicted in Equation (9):

(9) $$K=\int B_{u}^{T}cB_{u}dv$$

Dielectric conductivity:

(10) $$K_{d}=-\int B_{V}^{T}\varepsilon B_{V}dv$$

Piezoelectric coupling matrix:

(11) $$K_{z}=-\int B_{u}^{T}eB_{V}dv$$

where *K_d_* is dielectric conductivity, *K_z_* is piezoelectric coupling matrix, and F is a vector of nodal forces, surface forces, and body forces. The electrical load vector L is a vector of nodal surface and body charges.

### 3.2. Parametric Study of Cantilever Beam

A parametric study was conducted on the cantilevers’ length to analyze the impact of varying lengths (Section 3.2.1). During this study thickness of the beam was kept the equal to 0.45 mm (combined thickness of Nitinol with the Lithium niobate). A parametric sweep was also conducted to see how the acceleration (Section 3.2.2) can change the device’s voltage.

#### 3.2.1. Effect of the Length of the Cantilevers

Table 2 illustrates the six schematics of cantilever beams with varying lengths of L1, L2, L3, L4, L5, and L6. With the modal analysis conducted, the first four natural frequencies of each design are summarized in the table. Selecting the best geometry for the design of MPEH is strongly dependent on the frequency spectrum of the target vibration source being lower than 500 Hz. The final dimensions of the cantilever beam length are depicted in Table 3.

For designs 1,3,4 and 5, the first four resonance frequencies were below 250 Hz and for design 2 and 5, the first three resonance frequencies were below 250 Hz. Since the vibration source is expected to primarily operate below 500 Hz, it was important to do this kind of optimization to select the respective lengths of the cantilevers for effective device performance. 

It was worth mentioning that the design optimization of the geometry is mainly governed by the target frequency range. However, to reduce the resonance frequencies of the cantilever, further proof mass can be added to make it more compatible for random vibrational energy harvesting.

Each design’s first four natural frequencies are summarized in Table 2. This study has been conducted to select the best geometry for creating a multi resonant piezoelectric energy harvester. However, this will also depend on the source of excitation. In this research, our focus was mainly on a frequency lower than 500 Hz. That is why we targeted a frequency range of 1–500 Hz.

#### 3.2.2. Effect of the Acceleration on the Harvesting Device

**Figure 2:**
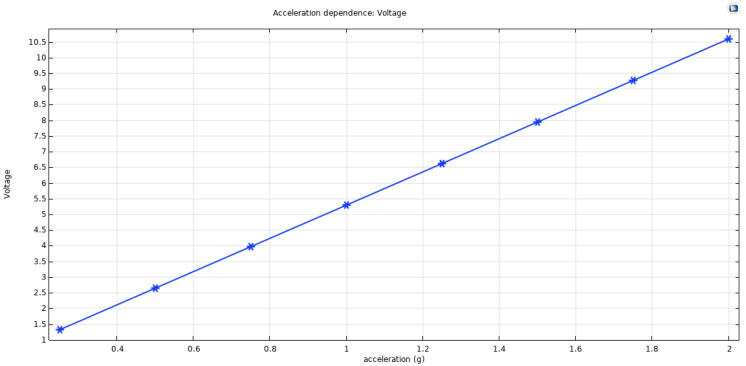
Evolution of the output voltage versus the acceleration.

We conducted another study to analyze the acceleration impact on the device; among others, we chose to work at 1 g acceleration. Still, it was essential to know how the device will work at high or low acceleration to check the device’s adaptability. The result is displayed in Figure 2, where we observed that an increase in the acceleration will increase the open-circuit voltage of the device. 

### 3.3. Modal and Harmonic Analysis of Multiresonant Piezoelectric Energy Harvester 

From the studies carried out in the previous section, we were able to select the desired cantilever lengths without any proof mass. With the geometry of the different lengths of beams, modal and harmonic analyses are further carried out in this section.

#### 3.3.1. Modal Analysis 

In the modal analysis of the cantilever beam, we chose the resonance frequency with high voltage values so that the first four frequencies for the six cantilevers would come under 500 Hz. Hereafter, we represented the resonance frequencies with high voltages for the multi resonant piezoelectric energy harvester summarized in Table 4.

**Figure 3:**
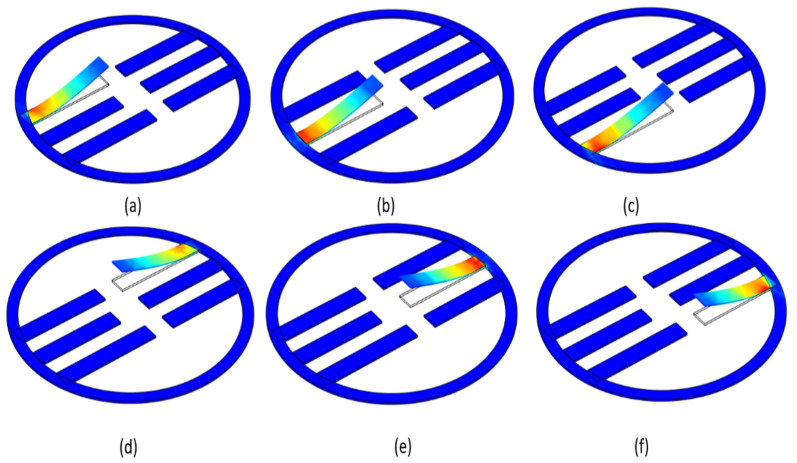
Deformation shapes of all the six cantilevers for the resonances having the maximum voltage the results obtained from COMSOL Multiphysics modal analysis. (**a**) Cantilever1; (**b**) cantilever 2; (**c**) cantilever 3; (**d**) cantilever 4; (**e**) cantilever 5; (**f**) cantilever 6.

The total deformation shapes (of three main axes) for the first six resonance frequencies corresponding to Table 4 are illustrated in Figure 3. Note that the red-colored section represents the maximum stress and maximum deformation. 

#### 3.3.2. Harmonic Analysis

**Figure 4:**
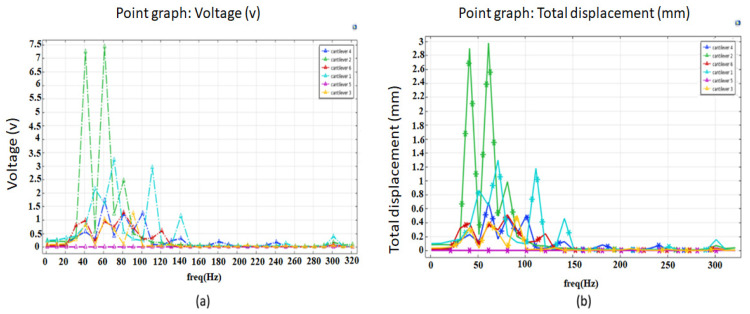
Frequency responses of multi resonant piezoelectric energy harvester (**a**) voltage and (**b**) displacement obtained from FEA under 1 g base excitation.

To verify the results we obtained from the modal analysis, we also performed a harmonic analysis. For this particular study, the excitation was maintained at 1 g. Henceforth, the frequency response was evaluated, where we calculated the voltage and displacement of the multi resonant piezoelectric energy harvester. Again, only a range of frequency between 1 Hz to 500 Hz was considered. The obtained results are presented in Figure 4a,b. As shown in Figure 4, maximum displacement and voltage were attained when the frequencies were closed to the resonance frequencies obtained from the modal analysis. This study indicates that the relation between the harmonic response analysis and modal analysis agrees closely.

**Figure 5:**
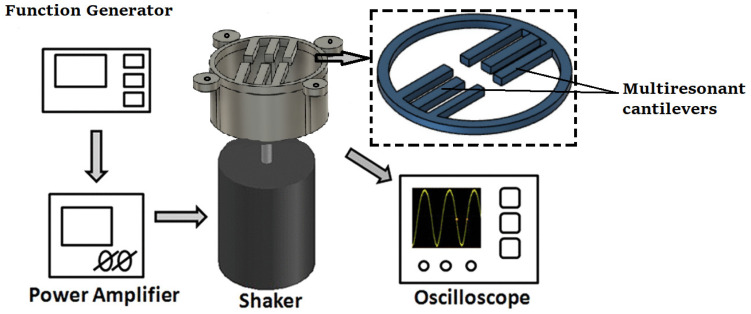
Schematic of the experimental setup for the multiresonant piezoelectric energy harvester.

The voltage against frequency response, as illustrated in Figure 5, shows similar behavior in which peak voltage is achieved at both resonance frequencies. We can see that at the resonance frequency, the cantilever (1) yields a maximum voltage of 3.3 V and the cantilever (2) produces 7.5 V, the remaining cantilevers (3), (4), (5), (6) yield a maximum voltage of 1.3 V, 1.7 V, 0.2 V, 1.3 V, respectively.

## 4. Experimental Validation

To validate the results, we obtained from the FEA simulation, an experimental study was carried out. The multiresonant piezoelectric energy harvester prototype was fabricated in a similar way to the previous section’s design, with all cantilevers having different sizes. As the prototype was made up of a metal substrate (Nitinol) in the cantilever beam’s shape, a lithium niobate layer was bonded over it. As we can see in Figure 5, the experimental setup consisted of a shaker used to produce the mechanical vibration. The shaker excited at the natural frequency of each cantilever and was driven by a sine wave from a function generator (RIGOL Technologies DG1022 20 MHz waveform generator; Starnberg, Germany).

**Figure 6:**
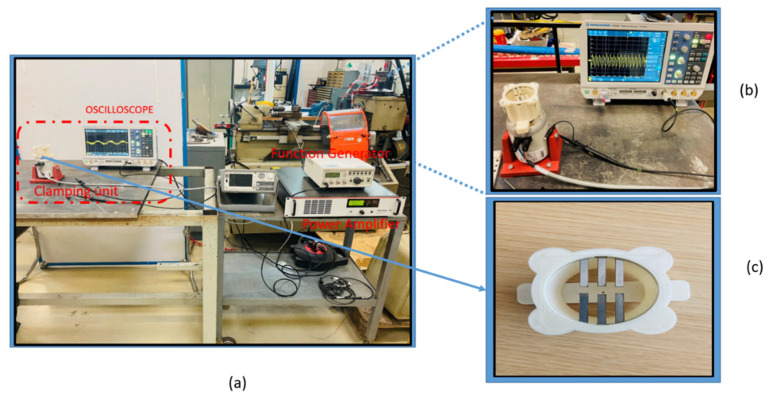
(**a**) The experimental setup (**b**) the fabricated E.H. prototype attached to the shaker (**c**) the fabricated prototype.

The output voltage was measured by an oscilloscope of four inputs. The acceleration was measured at the cantilever beam’s fixed base through an accelerometer (Dytran 3305A2, Chatsworth, CA, USA; 0.3 to 5000 Hz, ±5%). The acceleration and the voltage generated were recorded by NI DAQ modules, NI 9234 and NI 9229, respectively, through (Signal Express software of NI Company, Austin, TX, USA). The schematics of the experimental setup and the fabricated prototype are presented in Figure 6a–c, where the whole experimental design, along with a close view of the clamping unit with the shaker, are represented, respectively.

### 4.1. Response at Resonance

**Figure 7:**
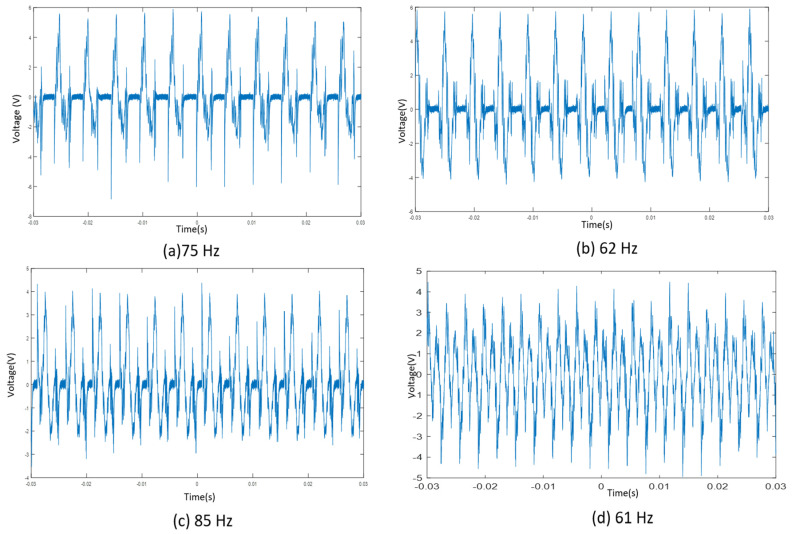

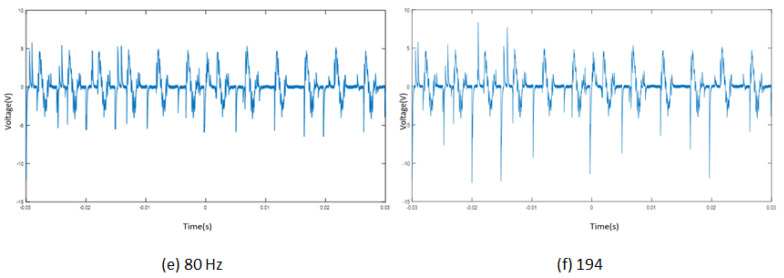
The output voltage of the multi resonant piezoelectric energy harvester at their respective resonance frequencies (**a**) third cantilever (**b**) fourth cantilever (**c**) fifth cantilever (**d**) firth Cantilever 4 (**e**) second cantilever (**f**) sixth cantilever.

Experiments were performed at the resonance frequency of each cantilever. Therefore, we could calculate and compare our results from the FEA simulation. the obtained results are displayed in Figure 7. It was found that at the resonance for the first cantilever, the output peak voltage for the system was 5 V_P,_ and for the second, third, fourth, fifth, and sixth cantilevers, the voltage was 4.5 V_P,_ 6 V_P_, 5.8 V_P_, 5.5 V_P.,_ and 1.5 V_P_, respectively. The third cantilever was the longest, and the sixth cantilever was the shortest in the following design.

### 4.2. Output Power at the Resonance Frequency

**Figure 8:**
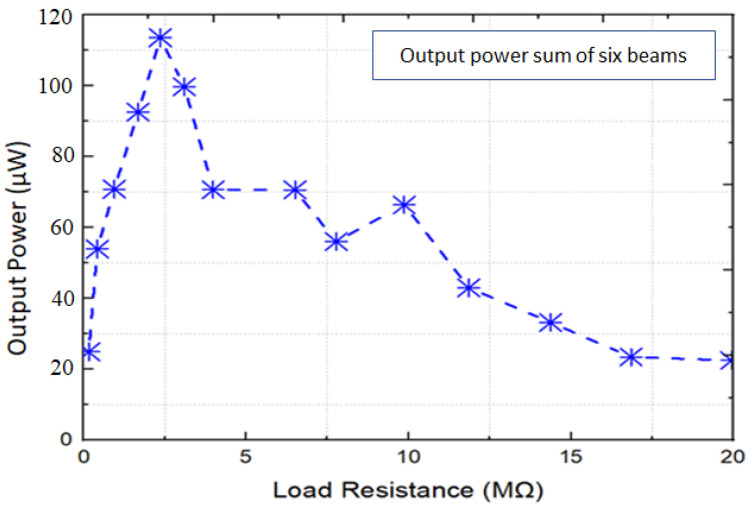
Power response from multimodal piezoelectric energy harvester for varying load resistances at 1 g base acceleration.

The output power of the MPEH at different load resistances was also calculated and depicted in Figure 8. The electrical load ranged from 1 MΩ to 20 MΩ, and the voltage response was measured at 1 g. As the electrical load increased, the voltage output from the harvester also increased monotonically. However, the power output reached peak values at 2.5 MΩ. Therefore, the harvester generated a useful power output for most of the frequency interval 21–71 Hz, with base accelerations as low as 1 g and an output power of 120 μW at their resonant peaks. 

### 4.3. Comparison of Experimental Results with Simulation Results

**Figure 9:**
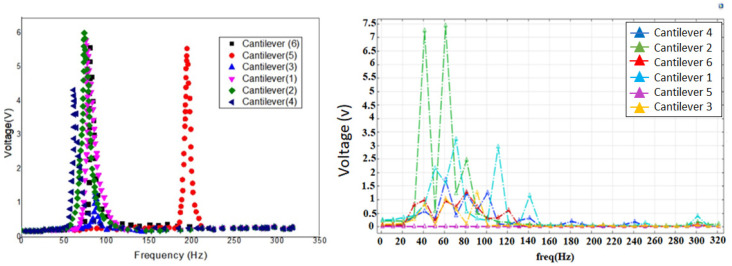
Graphical representation of the output voltage of the multi resonant piezoelectric energy harvester at their respective resonance frequencies (**a**) experimental (**b**) FEA simulation.

To validate the output voltage from the FEA simulation, we compared them with the FEA simulation results relative to the experimental results, see Table 5. In Figure 9, we present (a) experimental results and (b) FEA Simulation results. While the error is very small, it is worth mentioning that the resonance frequencies were similar in the experimental and simulation results. In conclusion, the experimental and the simulation results have certain differences due to the fillet added to provide support to the cantilever at the contact point with the circular ring. This behavior was dominantly seen in cantilevers 3 and 5. Moreover, due to these fillets, cantilevers 3 and 5 also experienced a comparatively greater damping effect than cantilevers 1, 2, 4 and 6, which is depicted in Figure 9a.

From Table 6, it is evident that the multiple resonating beam structure produces more output power in comparison to the single beam structure. Six beam cantilever structures had the advantage that their output power and bandwidth increased when the number of beams increased. Thus, such promising arrays enable the fabrication of well-functioning piezoelectric energy harvesters. Our structure has low output power in comparison to the structures listed in Table 6, as they are fabricated with lead-based material such as PZT, in comparison to the proposed design in this paper, which is based on lead-free materials. It is also paramount to compare the device sizes mentioned in Table 6 to better understand the output power. In [47], in which a prototype multimodal energy harvester with four piezoelectric elements was fabricated, a single piezoelectric element was able to generate peak power with a maximum of 249 µW, the dimension of the prototype length = 200 mm, width = 25 mm, thickness = 1.367 mm^.^ A polygon-shaped cantilever-based array is depicted, which employs the multifrequency operating principle [48]. The structure consists of eight cantilevers with an irregular design of the cross-sectional area. The cantilevers are connected to each other by specific angles to form polygon-shaped structures. The dimensions of the device consist of w_clamping_ = 35 mm, w_base_ = 5 mm, L_beam_ = 10 mm, W_fixing_ = 10 mm, D = 3.2 mm. 

## 5. Conclusions

This study shows that with lead-free materials, we can reduce our dependency on lead-based materials as the new materials are also capable of giving good performance. However, in this research, we showed how we could improve the performance of multiresonant piezoelectric energy harvesters based on lead-free material, lithium niobate, by implementing cantilevers of varying lengths and through the optimization of various design parameters. The presented multi resonant piezoelectric energy harvester can harvest energy from broadband, low frequency (60–250 Hz), and low amplitude ambient vibration sources. For the optimization and evaluation of the various parameters of the design, we used the FEA COMSOL Multiphysics tool. We conducted a parametric study using the same tool for the geometries with different lengths of the cantilevers and checked the resonance frequencies each of the cantilevers. With the presented design, we observed all six cantilevers having resonance frequencies under 1 g and excitation below 250 Hz. We also verified the simulation results with the experimental results. The results show that the proposed multi resonant piezoelectric energy harvester can work at an ambient vibration source as it performs under a low frequency range. It is worth mentioning that, for the first time, a multi resonant piezoelectric energy harvester based on lithium niobate has been presented within this paper.

It is recommended for future studies to conduct electrical interfacing to see the electrical response of this multi resonant piezoelectric energy harvester. We would like to point out that the high performance of these kinds of transducers cannot be attained by using bulk LiNbO_3_ wafers due to impedance–matching issues. Due to this reason, for bulk single crystals, the low value of the capacitance yields requires delicate interfacing with a typical electrical circuit. Another problem could be associated with matching electrical impedance to counter this problem. A specific electric circuit could be implemented so that these electric circuits can model the frequency response of supercapacitors and can work on high frequency. The results suggest that the faster the charge/discharge of this energy storage system, the lower the capacity value and, therefore, the lower the energy storage capability [50]. In addition, we can also reduce the thickness of the lithium niobate, which will increase our output and displacement. In this study, for simplicity purposes, we directly took the lithium niobate wafer at a thickness 0.35 mm, which can be factor that is improved in future studies. The use of a circular base can also potentially improve the design. Studies related to reducing the negative impact of antiresonance are also highly recommended.

## Figures and Tables

**Table 1:** Material properties of the multi resonant piezoelectric energy harvester (MPEH).

Parameter	Substrate (Cantilever Beam)	Piezoelectric
Material	Nitinol 45:55 (Ni:Ti)	Lithium Niobate (128° Y-cut)
Elastic modulus (GPa)	75–83	170
Poisson’s ratio	0.33	0.23
Density (kg/m^3^)	6450	4628
Piezoelectric constant (pC/N)	-	6–70
Capacitance (nF)	-	1.42
Thickness (mm)	0.10	0.35

**Table 2:** Representation of various geometries with different beam lengths and their natural (NF: natural frequency (Hz)).

Geometry	Cantilevers with Changed Length (mm)	Cantilevers with the Same Length (mm)	NF1	NF2	NF3	NF4
1.	21.5 (L1)	22 (L2, L3, L4, L5, L6)	51	61	101	221
2.	21 (L2)	22 (L2, L3, L4, L5, L6)	61	71	151	251
3.	24 (L3)	22 (L2, L3, L4, L5, L6)	21	31	61	101
4.	23 (L4)	22 (L2, L3, L4, L5, L6)	31	41	71	110
5.	22.5 (L5)	22 (L2, L3, L4, L5, L6)	41	51	81	121
6.	20.5 (L6)	22 (L2, L3, L4, L5, L6)	71	91	191	281

**Table 3:** Design parameter for the multiresonant piezoelectric energy harvester.

Description	Dimension (L × W × H)	Design Value (Units)
Outer circular ring	55 (dia)	mm
Inner circular ring	50 (dia)	mm
First cantilever	21.5 × 4 × 0.45	mm^3^
Second cantilever	21 × 4 × 0.45	mm^3^
Third cantilever	24 × 4 × 0.45	mm^3^
Fourth cantilever	23 × 4 × 0.45	mm^3^
Fifth cantilever	22.5 × 4 × 0.45	mm^3^
Sixth cantilever	20.5 × 4 × 0.45	mm^3^

**Table 4:** Natural frequencies for the MPEH (FEA) (NF: natural frequency).

Frequency	Hz
NF1	101
NF2	151
NF3	61
NF4	71
NF5	121
NF6	191

**Table 5:** Natural frequencies for the MPEH (FEA) (NF: natural frequency).

Cantilevers No.	FEA Resonance Frequency (Hz)	Experimental Resonance Frequency (Hz)	Error Percentage (%)
Cantilever 1	101	75	26
Cantilever 2	151	62	89
Cantilever 3	61	85	24
Cantilever 4	71	61	10
Cantilever 5	121	80	40
Cantilever 6	191	194	3

**Table 6:** Performance parameters comparison of different arrays of piezoelectric harvesters.

Work	No. Beams	Output Power	Bandwidth (Hz)	Value
[49]	3	1.1 Mw	39.5–44 (Hz)	mm
[47]	4	249 µW	10–20 (Hz)	mm
[48]	8	65.24 µW	10–240 (Hz)	mm
This Work	6	120 µW	21–71 (Hz)	mm
